# Case report: Vedolizumab in Oral Crohn’s disease: the downsides of a gut-specific therapy for a multi-site disease

**DOI:** 10.3389/fmed.2024.1485394

**Published:** 2024-12-03

**Authors:** Molly Harte, John Macken, Lifong Zou, Farida Fortune

**Affiliations:** ^1^Department of Oral Medicine, Royal London Hospital, Barts Health NHS Trust, London, United Kingdom; ^2^Centre for Oral Immunobiology and Regenerative Medicine, Institute of Dentistry, Faculty Medicine and Dentistry, Queen Mary University of London, London, United Kingdom; ^3^Centre for Oral Bioengineering, Institute of Dentistry, Faculty Medicine and Dentistry, Queen Mary University of London, London, United Kingdom; ^4^London Behcet’s Centre of Excellence, The Royal London Hospital, Barts Health NHS Trust, London, United Kingdom

**Keywords:** Crohn, oral medicine, vedolizumab, orofacial granulomatosis, gut selectivity

## Abstract

**Introduction:**

Crohn’s disease (CD) is a chronic inflammatory bowel disease which can affect any area of the gastrointestinal tract, including oral tissues. The complex nature of this disease demands interdisciplinary management, especially when both intestinal and oral manifestations are present.

**Case:**

This report presents the case of a 28-year-old male patient with oral, ileo-caecal and peri-anal CD managed jointly between Gastroenterology and Oral Medicine. Treatment with vedolizumab, an α4β7 integrin with gut-selective anti-inflammatory activity, resulted in excellent ileo-caecal disease control, but was ineffective in controlling oral manifestations. The absence of MAdCAM-1 expression in oral tissues, necessary for vedolizumab’s mechanism, meant that the drug’s anti-inflammatory effects were limited to the gut. This limitation led to worsening oral symptoms, necessitating concomitant azathioprine therapy to manage oral inflammation.

**Conclusion:**

Multidisciplinary collaboration is important when managing CD patients with both oral and gut involvement in CD. Clinicians should be aware that vedoluzimab may be beneficial for intestinal CD, but does not target inflammation in oral tissues due to its gut-specific action. Good knowledge of the pharmacology and mechanism of action of drugs prescribed can aid decision making when prescribing for this group of patients and can limit the need for polypharmacy, often associated with an increased adverse effect profile.

## Introduction

Crohn’s disease (CD) is a chronic, inflammatory bowel disease. The aetiopathogenesis of CD is not fully understood but is thought to involve a complex interplay between immune dysregulation, dysbiosis and environmental factors in a genetically susceptible patient (1), ultimately leading to focal areas of transmural inflammation affecting any area of the gastrointestinal tract, including the mouth (2).

Oral CD can present with oral ulceration and swellings of the face, lips and gingivae (2). Treatment for oral CD varies depending on the severity of disease and can range from dietary exclusion, topical mouthwashes including Triorasol (Quest Ltd.), topical and intralesional corticosteroids injections, to systemic corticosteroids and immunosuppression (2). The evidence base for efficacy of systemic therapies in patients with solely oral CD is limited (3) but in patients with both oral and intestinal CD, systemic immunosuppressants and biologic therapies initiated for the intestinal CD often prove beneficial in aiding management of the oral aspects of this disease. We report a case of a 28-year-old male patient with oral, perianal and ileocaecal CD treated with vedolizumab.

## Case report

The patient had been under the care of the oral medicine department since diagnosis of CD at age 13. His oral symptoms were initially well controlled with a combination of azathioprine (doses between 50-125 mg), a strict cinnamon and benzoate exclusion diet, and Triorasol (Quest Ltd.) mouthwash (betamethasone, nystatin and doxycycline). Despite good oral symptom control, the patient had refractory gut symptoms warranting biological response modifier therapy, prescribed by the gastroenterology department (Table 1). The patient’s compliance with long-term azathioprine therapy was poor and this was eventually stopped by the gastroenterology team in 2020. In 2022, after treatment failure with two anti-TNFα agents – adalimumab and infliximab - and an anti-IL12/IL-23 agent – ustekinemab, the patient was started on vedolizumab, an integrin antagonist.

**Table 1:** Timeline of systemic therapies used in the management of this patient’s Crohn’s disease.

Systemic agent	Target	Dates	Reason for cessation
Azathioprine	Purine synthesis	2011–2020	Poor compliance
Adalimumab	TNFα	2012–2014	Secondary loss of response
Infliximab	TNFα	2014–2018	Secondary loss of response
Ustekinemab	IL-12, IL-23	2019–2022	Primary non-response
Vedolizumab	Integrin α4b7	2022 - present	N/A

**Figure 1:**
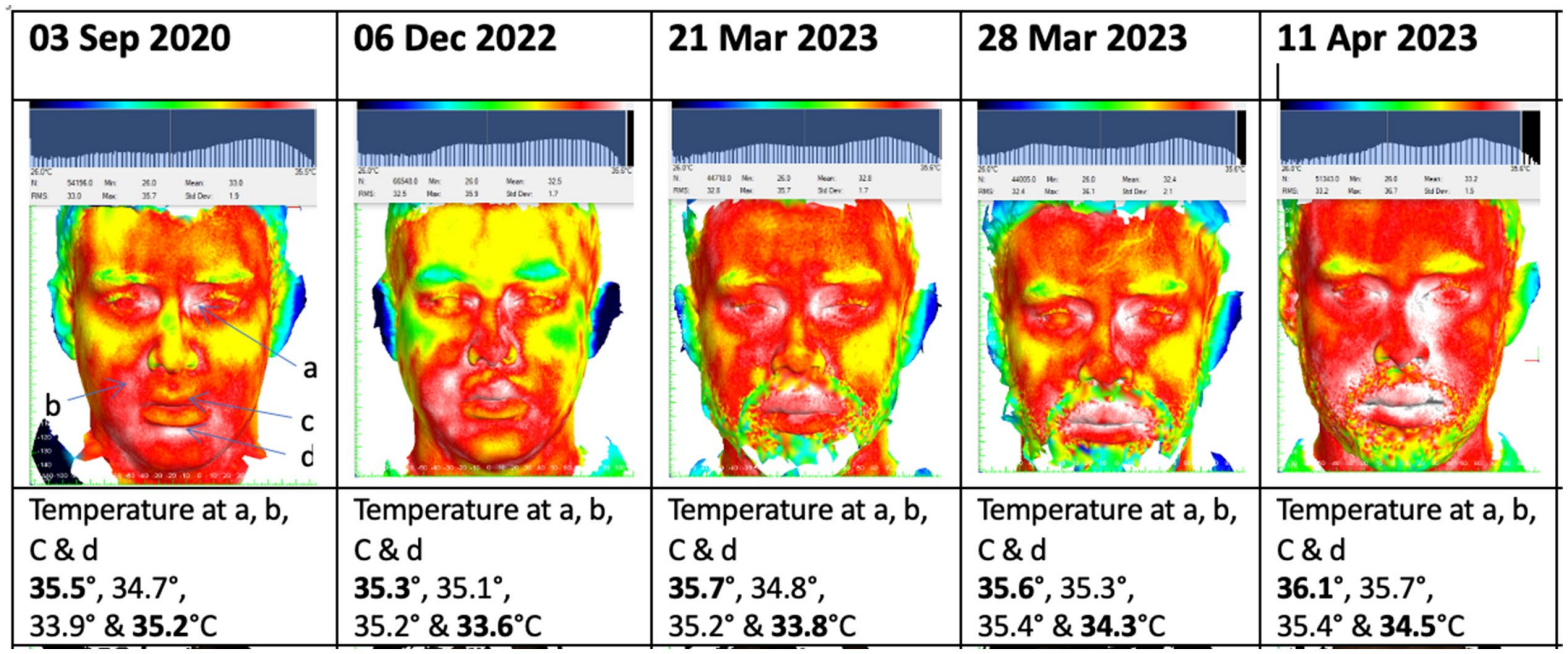
Sequential thermal images demonstrating progressively increasing facial temperature at 4 points following initiation of vedolizumab therapy in November 2022.

Within 2 months of vedolizumab therapy, the patient’s gut symptoms significantly improved. This was reflected in a reduction of his CRP from 119 mg/L to 4 mg/L. Despite this improvement, he started to experience a severe recurrence of oral symptoms with marked facial and lip swelling, deep linear oral ulceration and fibrotic banding in the buccal mucosae causing reduced mouth opening. Sequential thermal images (Figure 1) demonstrated a progressive rise in facial temperature which correlated clinically with increased facial swelling. Serum vedolizumab and anti-vedolizumab antibody testing was undertaken but demonstrated effective serum levels of vedolizumab with no vedolizumab specific antibodies. The oral symptoms were managed with increased use of Triorasol (Quest Ltd.) mouthwash and intralesional triamcinolone injections into areas of swelling, but the response was poor, and azathioprine was eventually re-initiated at 2 mg/kg to control the oral disease, alongside the vedolizumab with good effect.

## Discussion

The inflammatory response seen in CD is a complex T cell mediated process with increased levels of proinflammatory cytokines releasing IFN-*γ* and IL-12 as well as activation of TNFα, IL-1 and IL-6 (4). An important aspect of the immunopathology of CD is T cell migration from the blood to the intestinal tissue. This process involves integrins – leukocyte adhesion molecules. In CD, the α4β7 integrin binds to mucosal addressin-cell adhesion molecule 1 (MAdCAM-1) which is overexpressed in the intestinal mucosa of patients with active CD (5). The elaborate inflammatory processes seen in CD offer a range of potential therapeutic targets.

Anti-TNFα agents such as infliximab and adalimumab were the first biological response modifiers to be used in CD and remain a first-line biologic therapy for patients with moderate to severe CD (5, 6). They demonstrate good efficacy in both induction and maintenance of remission in intestinal CD (6, 7). The evidence base for the efficacy of anti-TNFα agents in oral CD specifically is largely limited to case reports where an anti-TNFα has been employed primarily for management of coexistant intestinal disease, but several studies have demonstrated good efficacy of infliximab and adalimumab for other extraintestinal manifestations of CD (8). Despite the efficacy of anti-TNF therapy, up to 40% of CD patients are primary non-responders, and a further 40% lose their response over time through the formation of anti-drug antibodies (9). This necessitates the use of alternative therapies, targeting different aspects of the complex CD inflammatory process.

The integrins are an alternative therapeutic target in CD. Natalizumab is a humanized monoclonal antibody which blocks both α4β7 and α4β1 (9). Blocking α4β7 inhibits its interaction with MAdCAM-1, thereby preventing lymphocyte trafficking to the intestinal mucosa. α4β1, however, binds to vascular adhesion molecule 1 (VCAM-1) which has a role in lymphocyte migration to the central nervous system. Blocking α4β1 has been associated with an increased risk of progressive multifocal leukoencephalopathy, limiting its use in CD (10).

**Figure 2:**
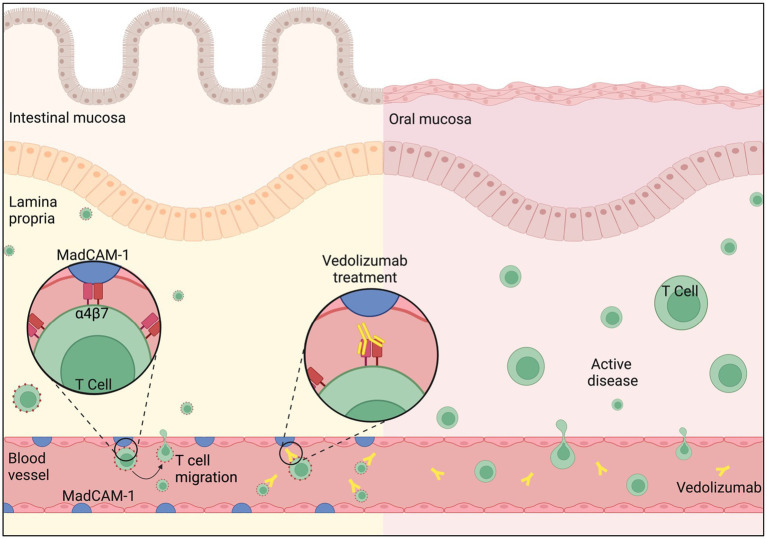
Intestinal mucosa: MAdCAM-1 (Mucosal Addressin Cell Adhesion Molecule-1) is primarily expressed on the endothelial cells of blood vessels in the intestinal mucosal tissue and is upregulated in CD. It binds to ‘Gut-homing T cells’, a specialized subset of T cells found in the gastrointestinal tract, via integrin α4β7 on the T cell surface. This binding mediates the adhesion and migration of T cells to sites of inflammation in the gut, triggering the Inflammatory cascade. Vedolizumab binds selectively to the α4β7 integrin, blocking its interaction with MAdCAM-1, and thereby preventing T cell translocation from the blood into inflamed intestinal mucosa. Oral Mucosa: MAdCAM-1 is not expressed in the oral mucosa. T cell trafficking and retention primarily relies on alternative adhesion molecules and chemokine receptors which drives disease activity in CD. Therefore, Vedolizumab does not prohibit T cell migration in the oral mucosal tissue. Figure created with BioRender.com.

Vedolizumab is a humanized monoclonal antibody against α4β7 integrin. It is licensed for use in moderately to severely active CD when initial therapy with anti-TNFα has failed, is not tolerated, or is contraindicated (11). A recent Cochrane review demonstrated good efficacy in induction and maintenance of remission in CD (12). Unlike natalizumab, which non-selectively binds to the α4 integrin subunit, vedolizumab binds selectively to the α4β7 integrin, inhibiting interaction with MAdCAM-1, and thereby preventing lymphocyte translocation from the blood into inflamed intestinal tissue (13). Given its demonstrated efficacy in the treatment of adult patients with moderate to severe CD and lower risk adverse effect profile when compared with nonselective α4 integrin inhibitors (14), it has become an increasingly popular treatment for patients who have not responded to anti-TNFα therapies. Despite the benefits of a gut-selective anti-inflammatory activity as produced by vedolizumab, the lack of MAdCAM-1 expression in the oral mucosa results in no clinical response in oral CD (Figure 2). This is demonstrated by the significant exacerbation of oral symptoms reported in our case, necessitating the use of additional intralesional corticosteroid and systemic immunosuppressant therapies for this patient.

While vedolizumab is often introduced for management of gastrointestinal CD following failure of anti-TNF therapies such as infliximab and adalimumab, other options for advanced therapies exist. Janus kinase (JAK) inhibitors, such as upadacitinib, offer a broader anti-inflammatory effect than vedolizumab, and therefore should be offered consideration in cases where the CD is not limited to the gut (15). Although there are currently no studies documenting the use of JAK inhibitors to treat Oral CD specifically, benefit has been reported with granulomatous cheilitis, which may be considered within the same disease spectrum (16).

Where immunosuppressant therapies, such as azathioprine, are required to be used alongside advanced therapies dosing adjustment of the immunosuppressant may be needed to minimize the risk of adverse effects. The immunosuppressant should be introduced gradually, with the dosing titrated according to patient response. Where azathioprine is used, thioguanine levels can be used to gauge optimal dosing (17).

CD may affect any area of the gastrointestinal tract, including the oral cavity, and management may overlap between gastroenterologists and oral medicine physicians. As biologic therapies become more targeted, consideration should be given to the patient’s disease phenotype prior to prescribing. Where oral CD exists concurrently with gastrointestinal CD, multidisciplinary discussion allows for treatment decisions to be made that are most appropriate for the patient’s disease experience. Further studies are needed to evaluate the efficacy of gut-selective therapies on the oral aspects of CD.

## Data Availability

The original contributions presented in the study are included in the article/supplementary material, further inquiries can be directed to the corresponding author.
